# Mechanisms of Cell Uptake and Transport of Xanthophylls in the Caco-2 Cell Model

**DOI:** 10.3390/nu18091389

**Published:** 2026-04-28

**Authors:** Fan Wu, Nan Chen, Yu Peng, Mo Li, Yuanying Ni, Tong Li, Ruihai Liu, Xin Wen

**Affiliations:** 1 College of Food Science & Nutritional Engineering, China Agricultural University, Beijing 100083, China; s20213061006@cau.edu.cn (F.W.);; 2 National Engineering Research Center for Fruit and Vegetable Processing, Beijing 100083, China; 3 Department of Food Science, Cornell University, Ithaca, NY 14853, USArl23@cornell.edu (R.L.)

**Keywords:** zeaxanthin, lutein, uptake, transporter, Caco-2 cell monolayer, endocytosis pathway

## Abstract

Background/Objectives: Zeaxanthin and lutein, which are essential dietary xanthophylls existing abundantly in free and esterified forms, require efficient intestinal absorption due to their insufficient synthesis in humans. However, limited knowledge on intestinal uptake and transport of xanthophyll esters is available. Methods: This study investigated the cellular uptake and transport mechanism of free and esterified xanthophylls using human Caco-2 cell monolayer, with lutein, zeaxanthin and their dipalmitates as representatives. Results: The results showed that free xanthophylls were uptaken without cellular re-esterification. Esterified xanthophylls were predominantly uptaken in free forms, as evidenced by Caco-2 cells incubated with zeaxanthin and lutein dipalmitates containing 80.8% and 89.4% of zeaxanthin and lutein, along with minor amounts of monoesters and diesters, respectively. Subsequent basolateral detection of both free xanthophylls and monoesters also confirmed intact ester uptake. Additionally, time- and concentration-dependent uptake patterns were observed, with all xanthophylls showing moderate permeability. Mechanistically, SR-BI and NPC1L1 were involved in the uptake of both free and esterified xanthophylls. At the expression level, free and esterified xanthophylls differentially affected ABCG5, with significant upregulation observed only in response to free xanthophylls. Tight junction integrity remained unaffected, excluding paracellular transport. Uptake of free and esterified xanthophyll micelles also involved clathrin- and caveolae-dependent endocytosis, whereas macropinocytosis was excluded. Conclusions: These findings provide insight into the uptake behavior of free and esterified xanthophylls and the transporter- and endocytosis-related processes involved.

## 1. Introduction

**Figure 1:**
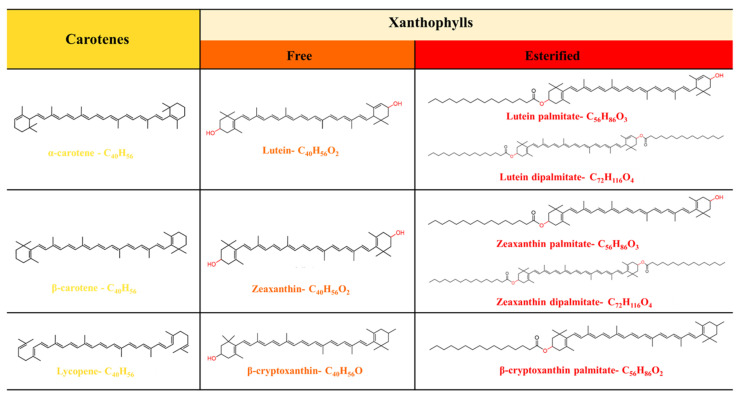
Classification and chemical structure of carotenoids.

Carotenoids are widely found in animals, plants, microorganisms, and algae [1], and can be divided into two main groups, carotenes (α-carotene, β-carotene, and lycopene) and xanthophylls (β-cryptoxanthin, zeaxanthin, and lutein), based on their structures (Figure 1). Xanthophylls are well known for their multiple physiologically active functions, including mitigating Alzheimer’s disease [2], improving memory and learning ability [3], and reduced risk of liver fibrosis and spinal cord injury [4]. Zeaxanthin (ZEA) and lutein (LUT) are two key xanthophylls known as “macular pigments”, which can cross the blood–retinal barrier and selectively accumulate in the macula and brain [5]. Frequent intake of ZEA and LUT could reduce the risk of eye diseases such as age-related macular degeneration [6,7,8] and improve cognitive function in humans [3]. Therefore, it is beneficial for humans to consume an adequate amount of xanthophylls from dietary sources.

Significant levels of xanthophylls can be found in certain fruits and flowers, such as goji berries (36 mg/100 g fresh weight) [9], red *Physalis* fruits (21 mg/100 g fresh weight) [10], and marigold (*Tagetes* sp.) (17–575 mg/100 g fresh weight) [11], making them excellent sources for xanthophyll extraction and supplement product development. Notably, xanthophylls in materials primarily exist in their esterified forms, such as zeaxanthin dipalmitate (ZDP) or lutein dipalmitate (LDP). However, given that high xanthophyll content does not necessarily translate to high uptake and absorption, it is essential to assess the cellular uptake and bioavailability of free and esterified xanthophylls.

The majority of previous studies reported that only free carotenoids have been observed in human intestinal cells, tissues, or serum [12,13,14]. In terms of esterified xanthophylls, they were assumed to be hydrolyzed into free forms by digestive lipase or brush border membrane enzymes before their absorption [15]. However, a few studies found that xanthophyll esters could also be detected in the skin [12], serum [16,17], or colostrum [18]. Given the possibility of re-esterification of free xanthophylls after their uptake [19], it remains undefined whether xanthophyll esters can be uptaken directly by intestinal cells.

After the uptake of xanthophylls, their bioavailability also depends on the transport pathways. Researchers initially identified passive diffusion as the mechanism for transporting free carotenoids [20]. Subsequently, several studies reported that some lipid transporters were involved in the transport process of free xanthophylls, suggesting the existence of facilitated diffusion during their intestinal transport process. For instance, transport of ZEA could be mediated by scavenger receptor class B type I (SR-BI) lipid transporter [21], while not by Niemann-pick C1-like1 (NPC1L1) cholesterol transporter [22]. Likewise, LUT was found to be transported by SR-BI [23,24], but not by NPC1L1 cholesterol transporter [22] and ATP-binding cassette (ABCG) transporter [23]. Nevertheless, there also have been a few studies with contradictory results. Sato [23] found that the selective chemical inhibitor of NPC1L1 cholesterol transporters significantly inhibited LUT absorption by Caco-2 cells, indicating the involvement of NPC1L1 cholesterol transporters in the uptake and transport of LUT. However, there is no information on the lipid transporters involved in the uptake and transport of esterified xanthophylls. Furthermore, it also remains unknown about the effect of free and esterified xanthophylls on intestinal tight junction (TJ) barrier function and cell membrane permeability, as well as whether they could be taken up by intestinal cells via the endocytic pathway.

Therefore, this study aimed to evaluate the uptake of xanthophylls (apical retention, cellular uptake, basolateral secretion, as well as time- and concentration-dependence uptake) and transport mechanism (transporters, paracellular and endocytic pathways) of free and esterified xanthophylls by using the Caco-2 cell monolayer. ZEA, LUT, and their corresponding diesters, ZDP and LDP, were studied as representatives. Our results indicated that esterified xanthophylls were mainly taken up in free forms, along with minor amounts of monoesters and diesters. Additionally, both free and esterified xanthophylls were transported by SR-BI and NPC1L1, as well as could be taken up through clathrin- and caveolae-mediated endocytic pathways. In contrast, free and esterified xanthophylls differentially affected ABCG5 expression, with significant upregulation observed only in response to ZEA and LUT. This study compared the uptake behavior of free and esterified xanthophylls and suggested the involvement of transporter- and endocytosis-related processes.

## 2. Materials and Methods

### 2.1. Materials

Authentic standards (≥95%) of (all-E)-lutein, (all-E)-lutein dipalmitate, (all-E)-zeaxanthin, and (all-E)-zeaxanthin dipalmitate were purchased from CaroteNature (Ostermundigen, Switzerland). Phosphatidylcholine, lysophosphatidylcholine, monoolein, oleic acid, cholesterol (water soluble), and sodium taurocholate were purchased from Sigma (Shanghai, China). Dulbecco’s modified Eagle’s medium (DMEM) (pH 7.2–7.4), trypsin-EDTA (0.25%), penicillin, streptomycin, and fetal bovine serum (FBS) were purchased from Gibco Life Technologies (Grand Island, NY, USA). The Caco-2 cell line was purchased from the American Type Culture Collection (ATCC) (Manassas, VA, USA). Transwell cell culture chamber inserts (Model 3401, 12 mm membrane diameter, polycarbonate membrane at the bottom of the inner chamber, membrane pore size 0.4 μm, membrane area 1.12 cm^2^) and 12-well plates were obtained from Corning (Corning, NY, USA). Rabbit polyclonal antibodies against β-actin (anti-β-actin), and NPC1L1 (anti-NPC1L1) were purchased from Cell Signaling Technology (Danvers, MA, USA). Rabbit polyclonal antibodies against SR-BI (anti-SR-BI) and ABCG5 (anti-ABCG5) were purchased from Santa Cruz (Heidelberg, Germany) and Absin (Shanghai, China), respectively. HRP-labeled goat anti-rabbit IgG (H+L) was obtained from Beyotime (Shanghai, China). Block lipid transport-1 (BLT-1), and ezetimibe were purchased from MedChemExpress (Monmouth Junction, NJ, USA). Sodium fluorescein, nystatin (caveolae (or lipid raft) inhibitor), dynasore (clathrin-mediated endocytic inhibitor), and 5-(N-ethyl-N-isopropyl)-amiloride (EIPA) (macropinocytosis inhibitor) were purchased from Aladdin (Shanghai, China). The alkaline phosphatase kit was obtained from Jiancheng Bioengineering Institute (Nanjing, China). Phosphate buffered solution (PBS), 96- and 6-well plates, and a BCA protein assay kit were purchased from Solarbio (Beijing, China). All other reagents were of analytical or HPLC grade.

### 2.2. Preparation of Synthetic Micelles

Synthetic mixed micelles were prepared using the method described by Reboul [24] and slightly modified. Amounts of 5.00 ± 0.20 μmol/L of ZEA, ZDP, LUT, or LDP, along with 0.04 mmol/L phosphatidylcholine, 0.16 mmol/L lysophosphatidylcholine, 0.3 mmol/L monoolein, 0.1 mmol/L free cholesterol, and 0.5 mmol/L oleic acid were mixed in chloroform–methanol (*v*/*v* 2:1) and stirred at approximately 60 rpm for 30 min. The mixed solution was then carefully evaporated under nitrogen purge and reconstituted in 15 mL of DMEM culture medium containing 5 mmol/L sodium taurocholate to prepare a reconstituted solution with a final concentration of 2.0 nmol/mL. The reconstituted solution was further sonicated (300 W, 37 °C) in a bath sonicator (Scientz-IID, Ningbo, China) for 2.5 min and centrifuged at 5000× *g* for 10 min. Finally, the micelles containing ZEA (ZEA-M), ZDP (ZDP-M), LUT (LUT-M), and LDP (LDP-M), were collected by filtering through 0.22 μm cellulose membranes (Klaus Ziemer, Mannheim, Germany), with average particle sizes of 104.03 ± 0.25 nm, 102.43 ± 3.65 nm, 103.37 ± 1.07 nm, and 104.73 ± 2.98 nm, respectively (Figure S1). Preliminary experiments confirmed that the maximum loading capacity of the micelles was approximately 30 nmoL, with a loading efficiency of about 95%. Prior to each experiment, the concentration of ZEA, ZDP, LUT, and LDP in the synthetic mixed micelles was measured to ensure a final concentration of 2.0 nmol/mL. The micelles were immediately used for uptake by Caco-2 cells.

### 2.3. Cytotoxicity

The cytotoxicity of ZEA-M, ZDP-M, LUT-M, and LDP-M against Caco-2 cells was evaluated by the Cell counting kit-8 (CCK-8) assay [25]. Briefly, Caco-2 cells (passage 10–20) were seeded at 1 × 10^4^ cells/well in a 96-well plate and cultured in an incubator (37 °C, 5% CO_2_) for 24 h. Then, the medium was replaced by the fresh medium containing micelles with different concentrations (diluted with DMEM medium at ratios of 1:0, 1:1, 1:3, 1:5, and 1:7, with corresponding xanthophyll concentrations of, ca., 2.00 nmol/mL, 1.00 nmol/mL, 0.50 nmol/mL, 0.33 nmol/mL, and 0.25 nmol/mL, respectively). After incubation for 24 h, the DMEM medium was discarded, 10 μL of CCK-8 solution was added to each well, and the cells were cultured in the incubator (37 °C, 5% CO_2_) for 1.5 h. The absorbance of each well was determined at 450 nm by a microplate reader (Spark, TECAN, Crailsheim, Germany). Cytotoxicity was calculated as the percentage of average treatments to the average controls, and cell viability equals 100% minus cytotoxicity%.

### 2.4. Establishment and Verification of Caco-2 Cell Monolayer Model

The Caco-2 cell monolayer model was established and verified [25] with minor modifications. Briefly, Caco-2 cells (passage 20–35) were seeded into transwell plates at a density of approximately 8.9 × 10^4^ cells/cm^2^ (equivalent to 2 × 10^5^ cells/mL, 0.5 mL) in the apical chamber, and 1.5 mL of cell-free complete medium was added in the basolateral chamber. The complete medium was changed every 2 days in the first week and then every day in the following two weeks. The Caco-2 cell monolayer with transepithelial electrical resistance (TEER) value (Millicell-ERS, Millipore, Bedford, MA, USA) above 500 Ω/cm^2^ was selected for subsequent uptake experiments. The alkaline phosphatase (AKP) activity was detected by an alkaline phosphatase kit to test whether the Caco-2 cell monolayer had differentiated. Furthermore, 0.5 mL of 2 mg/mL sodium fluorescein solution was added to the apical (AP) side to determine the integrity and transport function of the cell monolayer membrane.

### 2.5. Uptake by Caco-2 Cells

The ZEA-M, ZDP-M, LUT-M, and LDP-M were taken up by Caco-2 cells according to the previous method [26] with slight modifications. The micelles were mixed with DMEM medium at a ratio of 1:1 to prepare the uptake sample, with a corresponding xanthophyll concentration of 1.0 nmol/mL. To investigate the uptake of ZEA-M, ZDP-M, LUT-M, and LDP-M, 0.5 mL of each sample was received by the AP side of the Caco-2 cell monolayer and 1.5 mL DMEM medium by the basolateral (BL) side, then the cells were incubated (37 °C, 5% CO_2_) for 5 h to simulate human absorption time. The recoveries (%) of ZEA, ZDP, LUT, and LDP after incubation were tested to be 97.9%, 92.7%, 93.7%, and 94.1%, respectively. The apical retention, cellular uptake, and basolateral secretion were calculated as the proportion of the carotenoids presented at the AP side, in the cytoplasm, and at the BL side after uptake to those in the added mixed micelles, respectively. Uptake efficiency (%) referred to the percentage of carotenoids in both the cells and the DMEM medium from the BL side to those in the added mixed micelles.

### 2.6. Time- and Concentration-Dependent Uptake by Caco-2 Cells

The time- and concentration-dependent uptakes were explored by changing the incubation time and the concentration of xanthophyll micelles, respectively. Briefly, to investigate the time-dependent effect, the AP side of the Caco-2 cell monolayer received 0.5 mL of micelle sample and the BL side received 1.5 mL of DMEM medium. The cells were incubated (37 °C, 5% CO_2_) for different times (1 h, 2 h, 3 h, 4 h, 5 h, and 6 h). To investigate the concentration-dependent effect, the AP side of the Caco-2 cell monolayer received 0.5 mL of micelle sample with varying xanthophyll micelle concentrations (diluted with DMEM medium at a ratio of 1:1, 1:2, 1:3, 1:4, and 1:5, with corresponding xanthophyll micelle concentration of, ca., 1.00 nmol/mL, 0.67 nmol/mL, 0.50 nmol/mL, 0.40 nmol/mL, and 0.33 nmol/mL, respectively), and the BL side received 1.5 mL of DMEM medium; then the cells were incubated (37 °C, 5% CO_2_) for 5 h. All the transwell chambers were washed twice with 1 mL of PBS, then the cells were scraped into 1 mL PBS. At the same time, the DMEM medium from the AP side and the BL side was collected separately. All of the collected samples were stored at −80 °C before the analyses of carotenoids.

### 2.7. Measurement of Apparent Permeability Coefficient

The apparent permeability coefficients (P_app_) of ZEA, ZDP, LUT, and LDP in micelles across Caco-2 cell monolayers were measured using the method reported by Yu and Huang [27] and slightly modified. Briefly, for the transport from the AP side to the BL side (AP → BL), 1 mL of micelle sample was added at the AP side as the donor chamber, and 2 mL of DMEM medium was added to the BL side as the receiving side; in terms of the BL → AP transport, 2 mL of micelle sample was added at the BL side as the donor chamber, and 1 mL of DMEM medium was added at the AP side as the receiving side. The Caco-2 cell monolayers were cultured in the incubator (37 °C, 5% CO_2_), and 1 mL (AP → BL) or 0.5 mL (BL → AP) samples from the receiving side were aspirated at 30, 60, 90, and 120 min, respectively, with the same volume of DMEM medium replenished each time. The samples were stored at −80 °C before analyses of carotenoids. The P_app_ was calculated based on the total amount of carotenoids, including their detectable metabolites in the receiver compartment, using the following equation:

(1) $$P_{app}\left(\frac{cm}{s}\right)=\frac{dQ}{dt}\times \frac{1}{\left(A\times C_{0}\right)}$$

dQ/dt is the steady-state flux (μg/s); A is the membrane area (cm^2^); C_0_ is the initial concentration of carotenoids (μg/mL).

The efflux ratio (P_ratio_) was calculated according to the following equation:

(2) $$P_{ratio}=\frac{P_{app}(BL\to AP)}{P_{app}(AP\to BL)}$$

### 2.8. Transporters Involved in the Uptake Processes

#### 2.8.1. Western Blot Analysis of Transporter Expression

The transporters involved in the uptake and transport processes of ZEA, ZDP, LUT, and LDP were investigated following the Western blot method described by Li, Wei, Zhao, Yu, Huang and Li. [25] with slight modifications. Caco-2 cells (passage 20–35) in complete medium were seeded at 1.2 × 10^6^ cells/well in a 6-well plate and cultured in an incubator (37 °C, 5% CO_2_) for 48 h. After aspirating the complete medium, the cells received 2.5 mL of micelle sample, and were cultured in the incubator (37 °C, 5% CO_2_) for 24 h. After 24 h incubation, the cells were washed with cold PBS and lysed by adding 200 μL of cell lysis buffer (containing 194 μL RIPA strong lysate, 2 μL PMSF, 2 μL protease inhibitor, and 2 μL protein phosphatase inhibitor) per well on ice for 20 min with shaking to obtain the scraped cell samples. The scraped cell samples were sonicated at 100 W for 45 s and centrifuged at 14,000× *g* and 4 °C for 30 min to obtain the supernatant. The total protein content was determined according to the BCA kit method. The protein samples were separated by 10% SDS-polyacrylamide gel electrophoresis and transferred to the polyvinylidene fluoride (PVDF) membranes by wet transfer (at a constant current of 200 mA, 2 h) on ice. After blocking in the 37 °C incubator for 15 min with special blocking solution (Beyotime, Shanghai, China), the PVDF membranes were incubated at 4 °C in primary antibodies (anti-β-actin, anti-SR-BI, anti-NPC1L1, anti-ABCG5, anti-ZO-1, and anti-claudin-1; dilution ratio was 1:1000) with shaking overnight. Subsequently, the PVDF membranes were washed by PBS 3 times with 10 min each time and incubated with HRP-labeled goat anti-rabbit IgG (H+L) (1:2000 dilution) at room temperature for 1 h, then visualized with ECL Plus enhanced chemiluminescence (Beyotime, Shanghai, China). The membranes were detected by the Tanon 4600 chemiluminescence imaging system (Tanon, Shanghai, China). Data analysis was performed with ImageJ image analysis software (version 1.53q, National Institutes of Health, Bethesda, MD, USA).

#### 2.8.2. Assessment of Transporter Involvement Using Specific Inhibitors

The transporters involved in the uptake and transport processes of ZEA, ZDP, LUT, and LDP were investigated following the method previously described [23,28] with slight modifications. The cells were pretreated with scavenger receptor chemical inhibitors, i.e., 10 µmol/L BLT-1 and 40 µmol/L ezetimibe with DMEM medium, for 1 h. Then, the cells were washed with the PBS buffer twice and received 2.5 mL of xanthophyll micelle sample before being cultured in the incubator (37 °C, 5% CO_2_) for 24 h. After 24 h, the cells were scraped and collected in 1 mL of PBS and then stored at −80 °C before the analyses of carotenoids.

### 2.9. Identification of Endocytosis Pathways Using Inhibitors

The investigation of endocytosis pathways was performed following the detailed protocol described by Li, Wei, Zhao, Yu, Huang and Li. [25] and slightly modified. Three inhibitors, i.e., dynasore, EIPA, and nystatin, were weighed exactly and configured into 25 μg/mL solution with DMEM medium. The Caco-2 cell monolayers received 0.5 mL of inhibitor solution on the AP side and 1.5 mL of DMEM medium on the BL side and then were incubated in an incubator (37 °C, 5% CO_2_) for 30 min. After aspirating the liquid out of the AP side, 0.5 mL of micelle sample was added to the AP side and incubated for 4 h (37 °C, 5% CO_2_). Finally, the Caco-2 cells were scraped, and the DMEM medium from the BL side was collected and stored at −80 °C before the analyses of carotenoids.

### 2.10. Carotenoid Analyses

The concentrations of ZEA, ZDP, LUT, and LDP in cell samples and DMEM medium from the AP and BL sides were determined using the previous method [29] with minor modifications. The extraction solvent was prepared with methanol, ethyl acetate, petroleum ether (1:1:1, *v*/*v*/*v*), and 0.1 g/L BHA and BHT were added to avoid carotenoid oxidation during extraction. An amount of 1 mL of cell samples or the DMEM medium from the AP and BL sides was mixed with 12 mL of extraction solvent and then sonicated by a probe-sonicator (Scientz-IID, Ningbo, China) at 100 W for 60 s in the ice bath. After that, the mixture was centrifuged (3000× *g*, 10 min, 4 °C), the supernatant was collected, and the pellet was re-extracted by the extraction solvent 3–4 times until colorless. All the collected supernatants were combined afterwards to obtain the carotenoid extracts, which were dried under a gentle stream of nitrogen and stored at −80 °C. Before HPLC analyses, the dried extracts were dissolved in 2.0 mL of *tert*-butyl methyl ether (tBME) and methanol (1:1, *v*/*v*) and filtered (0.45 μm PTFE membrane) into brown vials.

The identification and the quantification of carotenoids were carried out by HPLC (LC-20A, Shimadzu, Kyoto, Japan). The carotenoids were separated on a C30 reversed-phase column (250 mm × 4.6 mm, i.d. 3 μm; YMC, Kyoto, Japan) protected by a YMC C30 guard column of the same material, under the following mobile phases with methanol/tBME/water (80:18:2, *v*/*v*/*v*) as eluent A and methanol/tBME/water (8:90:2, *v*/*v*/*v*) as eluent B. The elution gradient was as follows: from 0% to 30% B in 5 min, from 30% B to 86% B in 30 min, from 86% B to 100% B in 2 min, from 100% B to 0% B in 4 min, and isocratic at 0% B for 4 min. The total run time was 45 min at a flow rate of 1 mL/min. The column temperature was 40 °C, and the injection volume was 10 μL. Carotenoids were monitored at 450 nm. Free xanthophylls and their diesters were identified by comparison of retention times and UV/Vis absorption with their authentic standards and quantitated by linear external calibration of their corresponding standards. Monoesters of xanthophylls were identified by comparing their UV/Vis absorption by matching those of the free xanthophylls. As esterification of xanthophylls with fatty acids does not modify the conjugated chromophore or its light absorption properties [30]; monoesters were thus quantified using external calibration curves of authentic free xanthophyll standards. Molecular weight correction factors were applied. The limit of detection (LOD) and limit of quantitation (LOQ) were determined using the least-squares regression method based on the residuals of standards at each dilution level. Following the approach [31], the y-intercept was used to calculate the LOD (multiplied by 3) and LOQ (multiplied by 10). The LOD values for ZEA, ZDP, LUT, and LDP were determined to be 0.06, 0.01, 0.04, and 0.01 nmol/mL, respectively. Correspondingly, the LOQ values were calculated as 0.20, 0.03, 0.13, and 0.03 nmol/mL, respectively.

### 2.11. Statistical Analyses

All experiments were performed at least in triplicate. SPSS Statistics Version 25.0 (IBM, Armonk, NY, USA) was used for the statistical analyses. The data were displayed as the mean values ± standard deviations. Normality and homogeneity of variances were examined by Shapiro–Wilk’s and Levene’s tests (*p* < 0.05), respectively. Differences were assessed by analysis of variance (ANOVA) at a significant level of *p* < 0.05. To further analyze the results, Tukey’s Honestly Significant Difference (HSD) post hoc test was used for data sets with equal variances, while Tamhane’s test was applied for data sets with unequal variances.

## 3. Results and Discussion

### 3.1. Apical Retention, Cellular Uptake, and Basolateral Secretion of Free and Esterified Xanthophylls in Caco-2 Cell Monolayers

To determine the safe concentration range of the xanthophyll micelles that could ensure Caco-2 cell viability > 80% [25], the cytotoxic results of ZEA-M, ZDP-M, LUT-M, and LDP-M were evaluated by CCK-8 assays and are displayed in Figure S2. When the xanthophyll micelles were diluted with DMEM medium at ratios of 1:1, 1:3, 1:5, and 1:7, the cell viabilities were all greater than 90%. Therefore, the micelles were mixed with DMEM medium at a ratio of 1:1 to prepare the uptake sample with corresponding xanthophyll concentrations of 0.92 ± 0.06 nmol/mL for ZEA-M, 0.95 ± 0.08 nmol/mL for ZDP-M, 0.96 ± 0.02 nmol/mL for LUT-M, and 0.93 ± 0.01 nmol/mL for LDP-M, respectively. The micelles were added to the apical chamber, and the differentiated results of the Caco-2 cell monolayer are shown in Figure S3. The result suggested that TEER values between 18 and 21 days of incubation were above 500 Ω·cm^2^, and the AKP activity of the AP side was about three times higher than the BL side, which indicated that Caco-2 cell monolayers were differentiated to form a polarity along with tight junctions between cells. In addition, after incubation for 2 h, 29.6% of sodium fluorescein passed through the control transwell, but only 0.8% of sodium fluorescein passed through the cell monolayer, indicating that the Caco-2 cell monolayer model could maintain membrane integrity and therefore be used for subsequent xanthophyll uptake experiments.

**Figure 2:**
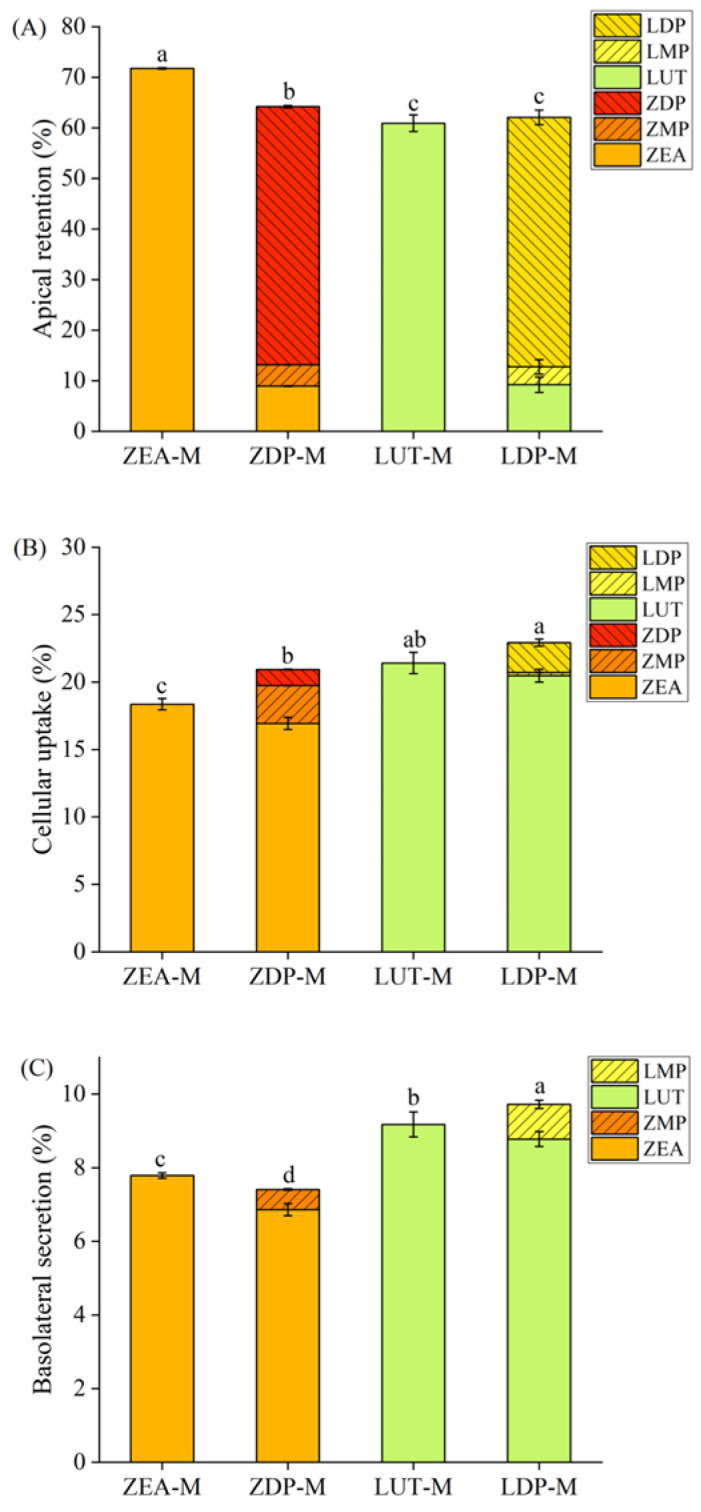
Percentages (%) of xanthophylls presented in the apical compartment (**A**), cytoplasm (**B**), and basolateral compartment (**C**) of differentiated Caco-2 cell monolayers after incubation with free and esterified xanthophyll micelles for 5 h. Data are mean ± SD, *n* = 3. Different lowercase letters indicated significant differences (*p* < 0.05) on apical retention, cellular uptake, and basolateral secretion of total xanthophylls from different micelles. The micelles were diluted with DMEM medium at a ratio of 1:1, yielding final xanthophyll concentrations of 0.92 ± 0.06 nmol/mL for ZEA-M, 0.95 ± 0.08 nmol/mL for ZDP-M, 0.96 ± 0.02 nmol/mL for LUT-M, and 0.93 ± 0.01 nmol/mL for LDP-M. ZEA-M, ZDP-M, LUT-M, and LDP-M are micelles of corresponding xanthophylls ZEA, ZDP, LUT, and LDP, respectively. ZEA: zeaxanthin; ZMP: zeaxanthin monopalmitate; ZDP: zeaxanthin dipalmitate; LUT: lutein; LMP: lutein monoplamitate; LDP: lutein dipalmitate.

To compare the uptake efficiency and composition of free and esterified xanthophylls absorbed by differentiated Caco-2 cell monolayers, the distribution of xanthophylls on the apical side, in the cytoplasm, and on the basolateral side was examined, respectively (Figure 2). On the apical side (Figure 2A), after 5 h of uptake by Caco-2 cell monolayers, the proportion of unabsorbed xanthophylls (i.e., retention on the AP side) from ZEA-M (71.7%) was significantly higher (*p* < 0.05) than that of ZDP-M (64.2%), while no significant difference (*p* > 0.05) was found between LUT-M (60.9%) and LDP-M (62.1%). It is worth noting that ZEA (8.9%) and zeaxanthin monopalmitate (ZMP) (4.2%) appeared in the unabsorbed zeaxanthin from ZDP-M. Similarly, the unabsorbed lutein from LDP-M contained LUT (9.2%) and lutein monopalmitate (LMP) (3.5%). On the one hand, the presence of free xanthophylls and their monoester could be probably attributed to the hydrolysis of ZDP or LDP by specific membrane enzymes of Caco-2 cells. Previous study uncovered that certain enzymes located on the brush border membrane, including diacylglycerol acyltransferase 1 (DGAT1) and acetyl-CoA, were involved in the hydrolysis of retinyl esters on the intestinal epithelial cells [15]. This suggests that there might have been similar brush border membrane enzymes responsible for the hydrolysis of ZDP and LDP as well. On the other hand, the intracellular free and esterified xanthophylls could be probably transported back to the AP side through retro-transport via some lipid transporters, such as SR-BI, which has been already found to participate in the uptake process of vitamin D [32] and vitamin E [33]. Therefore, corresponding experiments were also designed in this study to explore the transport characteristics of ZEA, ZDP, LUT, and LDP, in order to determine whether the retro-transport processes exist.

Regarding the cellular uptake, the proportion of xanthophylls absorbed from ZEA-M (18.4%) was significantly lower than that from ZDP-M (21.0%) (Figure 2B). This finding appeared to be inconsistent with previous reports by Chitchumroonchokchai and Failla [34], who reported that Caco-2 cells absorbed 17% of zeaxanthin from synthetic micelles containing only free zeaxanthin, while only 0.9% zeaxanthin was taken up from micelles containing solely zeaxanthin diesters. However, the absorption rate increased substantially to 12.4% when carboxyl ester lipase (CEL) was added. The discrepancy might be attributed to the different assessment times between their experiment (4 h) and ours (5 h). The additional hour likely allowed for greater hydrolysis of the carotenoid esters, leading to increased uptake. In terms of LUT-M and LDP-M, comparable uptake efficiencies of 21.4% and 22.8% were observed, respectively (Figure 2B). However, there was no relevant report previously on cellular uptake of lutein ester by Caco-2 cells. In addition, it was worth noting that free xanthophylls, xanthophyll monoesters, and xanthophyll diesters were all detected in Caco-2 cells incubated with ZDP-M or LDP-M, with ZEA, ZMP, and ZDP representing 80.8%, 13.4%, and 5.8% of the total cellular zeaxanthin, and LUT, LMP and LDP accounting for 89.4%, 1.0%, and 9.6% of the total cellular lutein, respectively. In the above-mentioned study by Chitchumroonchokchai and Failla [34], the absorbed 0.9% zeaxanthin in Caco-2 cells from synthetic ZDP micelles was entirely composed of ZDP. However, when CEL was added, the amounts and composition of accumulated zeaxanthin in cells changed, with ZEA, ZMP, and ZDP representing 69.0%, 27.0%, and 5.8% of the total absorbed zeaxanthin, respectively. Similarly, Dhuique-Mayer [35] found that only β-cryptoxanthin esters, but no free β-cryptoxanthin, was detected in Caco-2 cells after uptake of β-cryptoxanthin esters (β-cryptoxanthin laurate and β-cryptoxanthin myristate) from synthetic micelles. In our study, the presence of free xanthophylls and xanthophyll monoesters in Caco-2 cells incubated with ZDP or LDP likely resulted from the hydrolysis of these compounds by brush border membrane enzymes prior to uptake or by intracellular enzymes following uptake. Analysis of the unabsorbed fractions revealed only limited formation of hydrolysis products, indicating that extracellular hydrolysis before uptake was relatively minor. In contrast, intracellular xanthophyll profiles after incubation with ZDP-M or LDP-M were dominated by free xanthophylls and monoesters, suggesting that hydrolysis occurred predominantly after cellular uptake. Previous studies demonstrated that intestinal epithelial cells, including Caco-2 cells, express endogenous intracellular esterases, such as carboxylesterase 2 (CES2). These enzymes are capable of hydrolyzing lipophilic ester substrates following cellular uptake, providing a mechanistic basis for intracellular de-esterification [36,37]. Furthermore, the consistent observation in our study with the above research on detection of xanthophyll esters in Caco-2 cells incubated with xanthophyll ester micelles supported the possibility of direct uptake of xanthophyll esters by intestinal epithelial cells. Nevertheless, xanthophyll esters were not found in Caco-2 cells incubated with ZEA-M and LUT-M, suggesting a low likelihood of xanthophyll re-esterification. Sugawara and Yamashita [19] also observed that the re-esterification rate of ZEA was much lower compared to the more polar xanthophyll of peridinin in intestinal cells. It should be noted that the present study focused exclusively on the all-E isomers of lutein and zeaxanthin. However, xanthophylls may also occur as 9Z-, 13Z-, and other geometric isomers in natural matrices or after processing and storage. Since geometric isomerization may alter physicochemical properties, these isomers may exhibit different intestinal uptake efficiencies [38].

In terms of basolateral secretion, the proportion of secreted zeaxanthin from ZEA-M (7.79 ± 0.07%) significantly exceeded that from ZDP-M (7.50 ± 0.16%) (*p* < 0.05), while the content of secreted lutein from LDP-M (9.72 ± 0.33%) notably surpassed that from LUT-M (9.17 ± 0.31%) (*p* < 0.05) (Figure 2C). Interestingly, both free xanthophylls and xanthophyll monoesters were detected on the basolateral side of Caco-2 cells after incubation with ZDP-M and LDP-M. However, Chitchumroonchokchai and Failla [34] reported the absence of zeaxanthin esters in the basolateral compartment after absorbing synthetic micelles containing zeaxanthin esters. There were two possible explanations for the presence of xanthophyll monoester on the basolateral side from micelles of xanthophyll diesters, i.e., the direct secretion of monoesters from the cytoplasm, or the re-esterification of secreted free xanthophylls in the basolateral compartment. However, in cases of free xanthophyll-containing micelles, xanthophyll esters were not detected in the basolateral side, being consistent with the intracellular results. Therefore, the presence of monoesters is more likely due to intracellular de-esterification followed by secretion, rather than basolateral re-esterification. In enterocyte-like cells, such basolateral export of lipophilic compounds generally occurs in association with apoB-containing lipoprotein particles. Accordingly, the free xanthophylls and monoesters detected in the basolateral compartment were more likely secreted predominantly in a lipoprotein-associated form and chylomicron-like particles, rather than as freely dissolved molecules [39,40]. Nevertheless, the potential involvement of paracellular transport cannot be entirely ruled out and merits further investigation. Additionally, the possibility of re-esterification in the plasma could not be excluded. Granado [16] administered a lutein extract from marigold flowers (15 mg/d as mixed esterified forms) to 18 healthy volunteers for 4 months and observed traces of lutein monoester in participants’ serum for the first time. Similarly, Hempel [17] conducted a human study to compare the post-prandial bioavailability between ZEA and ZDP formulation and reported the transient post-prandial occurrence of ZDP from both formulations.

### 3.2. Effect of Incubation Time and Xanthophyll Micelle Concentration on Xanthophyll Uptake Efficiency

**Figure 3:**
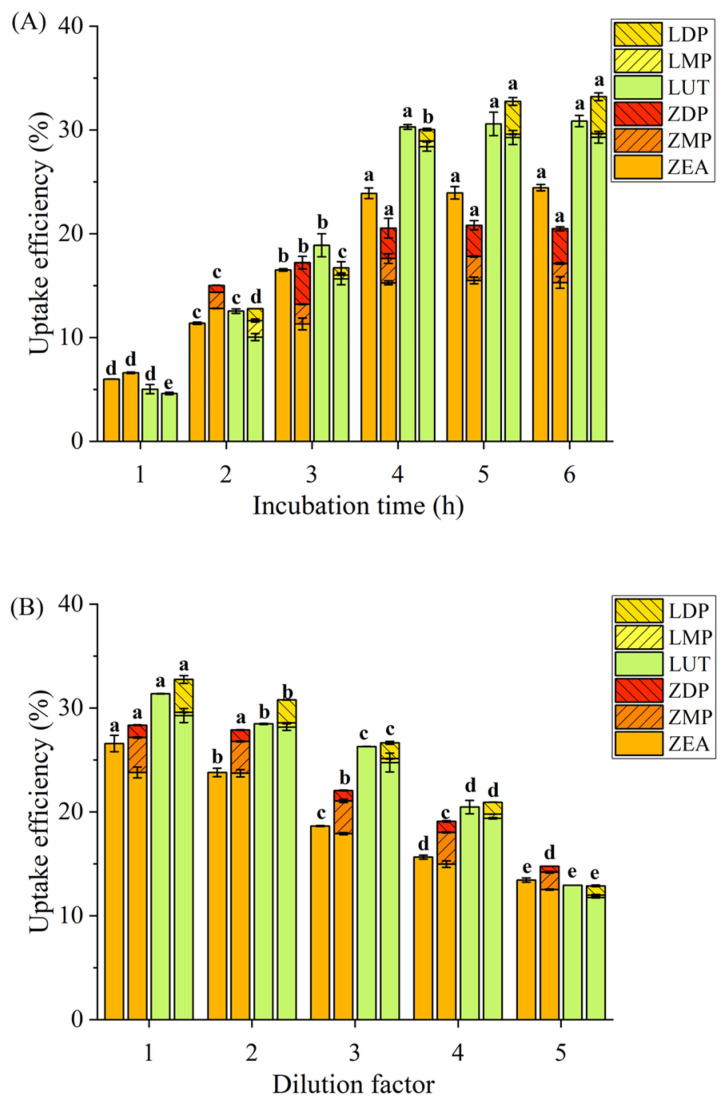
Effects of incubation time (**A**) and xanthophyll micelle concentration (**B**) on uptake efficiency (%) of xanthophylls after exposure to differentiated Caco-2 cell monolayers. Dilution factors of 1, 2, 3, 4, and 5 implied that the initial xanthophyll micelles were diluted by DMEM medium at ratios of 1:1, 1:2, 1:3, 1:4, and 1:5. Data are mean ± SD, *n* = 3. Different lowercase letters represented significant differences (*p* < 0.05) in the uptake efficiency of total xanthophylls at the different incubation times or dilution factors for the same micelle sample. ZEA-M, ZDP-M, LUT-M, and LDP-M were displayed from left to right at the same incubation time or micelle concentration, which represented micelles of the corresponding xanthophylls of ZEA, ZDP, LUT, and LDP, respectively. ZEA: zeaxanthin; ZMP: zeaxanthin monopalmitate; ZDP: zeaxanthin dipalmitate; LUT: lutein; LMP: lutein monopalmitate; LDP: lutein dipalmitate.

The effects of incubation time and xanthophyll micelle concentration on xanthophyll uptake in intestinal cells were evaluated to understand the response characteristics of these cells after xanthophyll administration. As depicted in Figure 3A, the uptake efficiency of xanthophylls from ZEA-M, ZDP-M, and LUT-M significantly increased (*p* < 0.05) with increasing incubation time from 1 h to 4 h, stabilizing thereafter at 5 h and 6 h. However, in terms of LDP-M, the maximum uptake was observed at 5 h, remaining constant at 6 h. While a concentration gradient was still present, the saturation of cellular transport machinery, feedback mechanisms due to intracellular accumulation, or possible micelle instability may have limited further uptake. Juan and Montesano [41] observed significant increases on uptake of LUT, ZEA, and ZDP by Caco-2 cells either from standard corresponding xanthophylls or from digested goji berries, comparing between those after 1 h incubation and those after longer incubation time of 2, 3, 4 h. However, only ZEA exhibited a time-dependent increase from 1 h to 4 h, aligning with our findings for ZEA-M, ZDP-M, LUT-M, and LDP-M. Interestingly, in a human study, Yao [42] reported peak concentrations of plasma lutein and zeaxanthin appeared between the 4th and 6th hours postprandially. While this might suggest comparable uptake windows between in vitro and in vivo settings, it is important to note that systemic absorption in humans is governed by more complex physiological factors, including ATP-dependent transport, enzyme activity, and homeostatic regulation. Therefore, the in vivo T_max_ should not be directly interpreted as equivalent to membrane transport kinetics observed in vitro. Furthermore, xanthophyll esters were undetectable in both the Caco-2 cell and on the basolateral side after 1 h incubation with ZDP-M and LDP-M. However, they became apparent with an incubation time exceeding 2 h, indicating the hydrolysis of xanthophyll esters upon reaching the apical membrane of intestinal cells, with most taken up in their free forms and a few accumulated as esters within the cells over time. In addition, no esterified xanthophyll was detected either in Caco-2 cells or on the basolateral side along 6 h incubation with ZEA-M and LUT-M, suggesting that free xanthophylls taken up were not re-esterified in Caco-2 cells, consistent with the results shown in Figure 2.

Figure 3B illustrated a decreased trend in xanthophyll uptake from ZEA-M, ZDP-M, LUT-M, and LDP-M with increasing dilution factors (1, 2, 3, 4, and 5). These findings suggested that the uptake and transport of free and esterified xanthophylls might involve more than just unidirectional or passive diffusion but also reversed or facilitated diffusion mechanisms, highlighting the necessity for further investigation on transport mechanisms.

### 3.3. Apparent Permeability Coefficient

The apparent permeability coefficient (P_app_) quantifies the instantaneous transfer of carotenoids, serving as an indicator of their uptake capacity. According to international standards, a P_app_ value higher than 1.0 × 10^−5^ cm/s signifies uptake ranging from 70% to 100%, indicating high uptake; a value between 1.0 × 10^−6^ and 1.0 × 10^−5^ cm/s suggests a 20% to 70% absorption rate, indicating moderate uptake; a P_app_ value below 1.0 × 10^−6^ cm/s indicates poor uptake [43]. This study conducted bidirectional transport experiments (AP → BL and BL → AP) to assess the uptake capacity of both free and esterified xanthophylls. The results revealed that P_app_ (AP → BL) and P_app_ (BL → AP) for all xanthophyll micelle samples were between 1.0 × 10^−6^ and 1.0 × 10^−5^ cm/s (Table 1), indicating moderate uptake of the corresponding free or esterified xanthophylls in Caco-2 cells. Interestingly, no significant difference (*p* > 0.05) was observed in P_app_ (AP → BL) of four kinds of xanthophyll micelles, while significant differences (*p* < 0.05) were found in P_app_ (BL → AP), with the efflux effect of free xanthophylls being superior to that of xanthophyll esters, and that of zeaxanthin more pronounced than lutein. Sato [23] reported P_app_ (AP → BL) and P_app_ (BL → AP) for lutein across the Caco-2 cell membrane were 5.0 × 10^−6^ cm/s and 2.1 × 10^−6^ cm/s, respectively, indicating moderate permeation of lutein, being consistent with our result for LUT-M.

The micelles were diluted with DMEM medium at a ratio of 1:1, yielding final xanthophyll concentrations of 0.92 ± 0.06 nmol/mL for ZEA-M, 0.95 ± 0.08 nmol/mL for ZDP-M, 0.96 ± 0.02 nmol/mL for LUT-M, and 0.93 ± 0.01 nmol/mL for LDP-M. Data are mean ± SD, *n* = 3. Different letters represented significant differences (*p* < 0.05) in the same column. Papp: apparent permeability coefficient. Pratio: trans-shipment ratio of Papp (BL → AP)/Papp (AP → BL). AP → BL: from the apical compartment to the basolateral compartment of Caco-2 cell monolayer model. BL → AP: from the basolateral compartment to the apical compartment of Caco-2 cell monolayer model. ZEA-M, ZDP-M, LUT-M, and LDP-M are xanthophyll micelles containing zeaxanthin, zeaxanthin dipalmitate, lutein, and lutein dipalmitate, respectively.

The efflux ratio (P_ratio_) was determined to estimate the existence of efflux or facilitated uptake, which was defined as the ratio of efflux permeability (BL → AP) and absorptive permeability (AP → BL). When Pratio exceeds 2, an efflux mechanism is implied; a P_ratio_ below 0.5 suggests the presence of a facilitated uptake mechanism; P_ratio_ between 0.5 and 2 indicates primarily passive diffusion as the uptake mechanism [27]. As shown in Table 1, the P_ratio_ values for ZEA-M, ZDP-M, LUT-M, and LDP-M fell in the 0.5 to 2 range, with no significant difference (*p* < 0.05) between free and esterified xanthophylls. It is possible, therefore, that the uptake mechanisms of both free and esterified xanthophylls were primarily passive diffusion, with potential involvement of facilitated diffusion and endocytic pathways.

### 3.4. Transporters of Xanthophylls in Caco-2 Cell Monolayer

Although passive diffusion possibly predominated in the uptake and transport mechanisms of free and esterified xanthophylls indicated by the above efflux ratio result, facilitated diffusion might also play a significant role in intestinal uptake. Previous studies also indicated that carotenoids interact with specific membrane lipid transporters for transport, including SR-BI, CD36, NPC1L1, and possibly the ATP-binding cassette (ABC) family [23,44]. However, these studies have predominantly focused on membrane lipid transporters that assist in the transport of free xanthophylls, leaving a gap knowledge concerning esterified xanthophylls. Furthermore, even for free xanthophylls, results have been inconsistent [21,22,23,24]. Therefore, this study aimed to investigate the specific membrane lipid transporters involved in the uptake process of both free and esterified xanthophylls.

#### 3.4.1. Role of SR-BI on the Apical Transport of Xanthophylls

**Figure 4:**
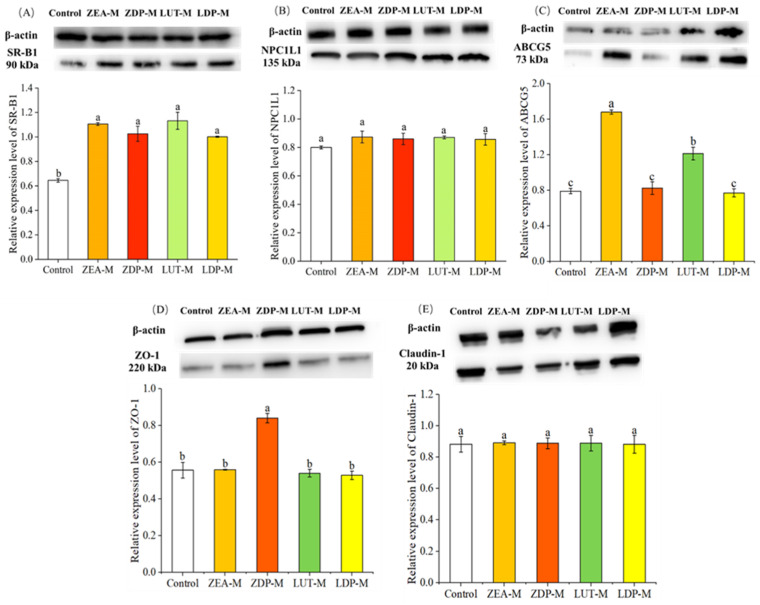
Effects of ZEA-M, ZDP-M, LUT-M, and LDP-M exposure on the expression of transporters in Caco-2 cells for 24 h. Representative band densities in Western blots and relative expression levels of SR-BI (**A**), NPC1L1 (**B**), ABCG5 (**C**), ZO-1 (**D**), and claudin-1 (**E**). Data are mean ± SD, *n* = 3. The micelles were diluted with DMEM medium at a ratio of 1:1, yielding final xanthophyll concentrations of 0.92 ± 0.06 nmol/mL for ZEA-M, 0.95 ± 0.08 nmol/mL for ZDP-M, 0.96 ± 0.02 nmol/mL for LUT-M, and 0.93 ± 0.01 nmol/mL for LDP-M. Different lowercase letters indicated significant differences (*p* < 0.05) among groups. Control: group without any treatment; ZEA-M, ZDP-M, LUT-M, and LDP-M are groups treated with xanthophyll micelles containing zeaxanthin, zeaxanthin dipalmitate, lutein, and lutein dipalmitate, respectively.

**Figure 5:**
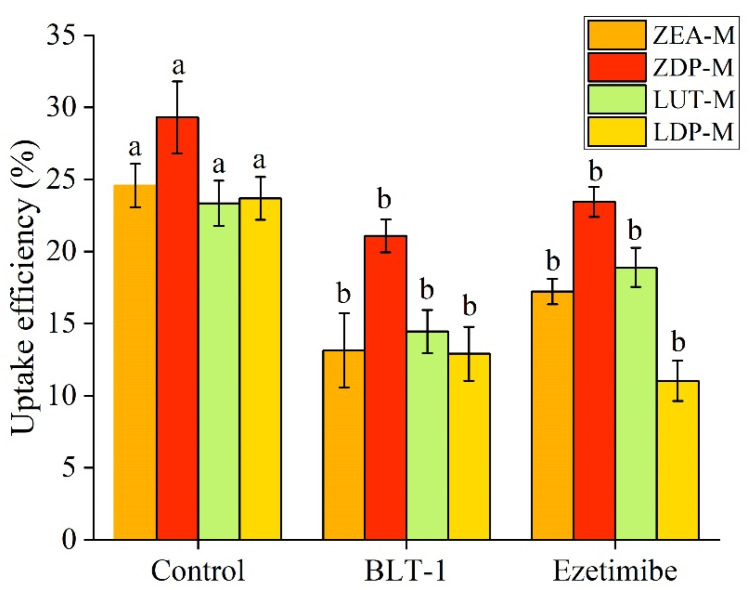
Effect of inhibitors (BLT-1 and ezetimibe) on uptake efficiencies (cellular uptake + secretion into the basolateral medium) of xanthophylls from ZEA-M, ZDP-M, LUT-M, and LDP-M into Caco-2 cells for 1 h. The micelles were diluted with DMEM medium at a ratio of 1:1, yielding final xanthophyll concentrations of 0.92 ± 0.06 nmol/mL for ZEA-M, 0.95 ± 0.08 nmol/mL for ZDP-M, 0.96 ± 0.02 nmol/mL for LUT-M, and 0.93 ± 0.01 nmol/mL for LDP-M. Different lowercase letters represented significant differences (*p* < 0.05) in the uptake efficiencies of xanthophylls from the same micelle when subjected to various inhibitors. Control: group without any inhibitor treatment. BLT-1: block lipid transport-1. ZEA-M, ZDP-M, LUT-M, and LDP-M are micelles of corresponding xanthophylls zeaxanthin, zeaxanthin dipalmitate, lutein, and lutein dipalmitate, respectively.

SR-BI, a multi-ligand transporter and class B scavenger receptor, has been implicated in carotenoid uptake and apical transport [21]. As depicted in Figure 4A, the relative expression levels of SR-BI in the ZEA-M, ZDP-M, LUT-M, and LDP-M groups were significantly enhanced (*p* < 0.05) compared to the control group, indicating its possible involvement in the intestinal uptake of ZEA, ZDP, LUT, and LDP. The involvement of SR-BI in the apical transport of xanthophyll was further investigated by examining the effect of block lipid transport 1 (BLT-1), a specific chemical inhibitor of SR-BI on xanthophyll uptake. As illustrated in Figure 5, the results demonstrated that BLT-1 significantly inhibited (*p* < 0.05) the uptake of ZEA, ZDP, LUT, and LDP, with inhibition rates of 46%, 28%, 38%, and 45%, respectively. These findings support the involvement of SR-BI in the apical transport of ZEA, ZDP, LUT, and LDP. Similar results were also reported by Sato [23], who found that BLT-1 inhibited free lutein accumulation in Caco-2 cells.

#### 3.4.2. Role of NPC1L1 on the Apical Transport of Xanthophylls

NPC1L1 has been established as a crucial factor in intestinal cholesterol absorption. The effects of free and esterified xanthophyll micelle exposure on relative expression levels of NPC1L1 were determined, but the results showed that there was no significant difference (*p* > 0.05) among groups (Figure 4B). These findings suggested that NPC1L1 expression was not significantly altered by xanthophyll exposure. However, ezetimibe, an inhibitor of NPC1L1, significantly inhibited (*p* < 0.05) the uptake of ZEA, ZDP, LUT, and LDP, with inhibition rates of 30%, 20%, 19%, and 53%, respectively, as depicted in Figure 5. This result indicated that NPC1L1 was involved in the apical transport of both free and esterified xanthophylls in this study. In line with this observation, Sato [23] reported that ezetimibe inhibited up to 40% of free lutein accumulation in Caco-2 cells, albeit they attributed this result to the inhibition of NPC1L1 expression.

#### 3.4.3. Effect of Free and Esterified Xanthophylls on ABCG5 Expression

ABCG5, a member of the sub-family G of ABC family, functions as an efflux protein that utilizes ATP to provide energy for transportation. ABCG5 forms a heterodimeric complex with ABCG8 that mediates sterol efflux at the apical membrane [45]. Coordinated transcriptional regulation of ABCG5 and ABCG8 in the intestine and liver has been reported in response to nutritional and metabolic perturbations [46]. Accordingly, changes in the expression of a single subunit can serve as an informative indicator of alterations in the activity state of the intestinal efflux machinery. Additionally, recent research has revealed the involvement of ABCG5 in the efflux processes of vitamin D, thereby regulating the absorption kinetics of dietary vitamin D [47]. This suggests a potential function of ABCG5 in the excretion of other fat-soluble substances by intestinal cells. While its specific role in carotenoid efflux remains to be elucidated, previous study indicated that polymorphisms in the ABCG5 gene may influence individual responses to lutein supplementation in humans [48]. Based on this evidence, the current study evaluated the expression level of ABCG5 on the efflux processes of ZEA-M, ZDP-M, LUT-M, and LDP-M. The results revealed significantly elevated ABCG5 expression levels in ZEA-M and LUT-M groups compared to the control group (*p* < 0.05), whereas no significant differences were observed among the ZDP-M, LDP-M, and control groups (*p* > 0.05) (Figure 4C). These findings revealed that ABCG5 expression was significantly elevated in response to ZEA and LUT, whereas no significant changes were observed in the ZDP and LDP.

Overall, the differential responses of SR-BI, ABCG5, and NPC1L1 at the expression level further suggest that xanthophyll exposure may selectively modulate intestinal transport-related proteins.

### 3.5. Role of Paracellular Pathways on the Transport of Xanthophylls

One of the primary functions of intestinal epithelial cells is to maintain a tight junction barrier, preventing the diffusion of external substances into the tissues through paracellular pathways. The expressions of ZO-1 and claudin-1 play crucial roles in regulating the tight junction barrier function. Significant decreases in ZO-1 and claudin-1 expression levels, coupled with reduced TEER values during carotenoid uptake, suggest the involvement of either transcellular or paracellular pathways [26]. However, in our experiments, no temporary reduction in TEER value was observed. As depicted in Figure 4D,E, there was no significant decrease (*p* > 0.05) in ZO-1 and claudin-1 expression levels for ZEA-M, ZDP-M, LUT-M, and LDP-M compared to the control group. These findings indicated that ZEA-M, ZDP-M, LUT-M, and LDP-M did not disrupt the integrity of the tight junction. While a minor degree of baseline paracellular permeability could not be fully excluded, the lack of TEER reduction or junctional protein suppression, along with the fact that the particle sizes of these micelles exceeded the pore size threshold for paracellular diffusion [49,50,51], suggested that paracellular transport was unlikely to be a significant pathway in this study.

### 3.6. Endocytosis Pathways of Xanthophylls in Caco-2 Cell Monolayer

**Figure 6:**
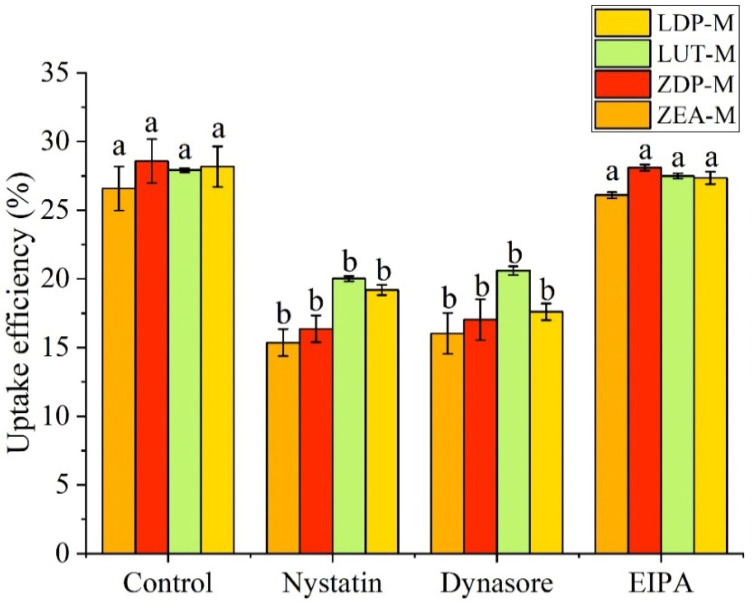
Effects of inhibitors (nystatin, dynasore, EIPA) on uptake efficiencies (cellular uptake + secretion into the basolateral medium) of xanthophylls from ZEA-M, ZDP-M, LUT-M, and LDP-M into Caco-2 cells for 4 h. The micelles were diluted with DMEM medium at a ratio of 1:1, yielding final xanthophyll concentrations of 0.92 ± 0.06 nmol/mL for ZEA-M, 0.95 ± 0.08 nmol/mL for ZDP-M, 0.96 ± 0.02 nmol/mL for LUT-M, and 0.93 ± 0.01 nmol/mL for LDP-M. Data are mean ± SD, *n* = 3. Different lowercase letters indicated significant differences (*p* < 0.05) on uptake efficiencies of xanthophylls from the same xanthophyll micelle after treated by different inhibitors. Control: group without any inhibitor treatment. EIPA: 5-(N-ethyl-N-isopropyl)-amiloride. ZEA-M, ZDP-M, LUT-M, and LDP-M are micelles of corresponding xanthophylls zeaxanthin, zeaxanthin dipalmitate, lutein, and lutein dipalmitate, respectively.

Nanoparticles can interact with the plasma membrane and enter intestinal cells via various endocytic pathways, including macropinocytosis, clathrin-mediated endocytosis, and caveolae-mediated transport [52]. Previous research on endocytosis pathways mainly focused on oral drugs or macromolecular substance delivery systems, but a recent study found that carotenoid-embedded nanoparticles could be directly taken up by intestinal cells [53], hinting at the potential involvement of endocytic pathways in carotenoid uptake. Therefore, this study investigated the contribution of different endocytosis mechanisms to the uptake of micellized free and esterified xanthophylls, using inhibitors including nystatin (a caveolae/lipid raft inhibitor), dynasore (a clathrin-mediated endocytic inhibitor), and EIPA (a macropinocytosis inhibitor). As depicted in Figure 6, treatments with nystatin and dynasore significantly reduced (*p* < 0.05) the uptake efficiency of ZEA-M, ZDP-M, LUT-M, and LDP-M compared to the control group. However, no significant differences (*p* > 0.05) were observed between the EIPA treatment group and the control group for all samples. These findings provide novel insights that xanthophyll micelles could also be taken up by intestinal cells via endocytosis, primarily through clathrin- and caveolae- or lipid raft-dependent routes.

## 4. Conclusions

Using zeaxanthin, lutein, and their corresponding diesters as representative compounds, this study comparatively characterized the uptake and transport behavior of free and esterified xanthophylls. The findings revealed that ZEA and LUT remained unesterified in both the cytoplasm and basolateral side, while ZDP and LDP were primarily taken up as free forms along with some monoesters and diesters, highlighting the possibilities of efficient enzymatic hydrolysis of the esters by brush border membrane enzymes in Caco-2 cells. A time- and concentration-dependent process across Caco-2 cells of free and esterified xanthophylls was found, and moderate uptake by Caco-2 cells was indicated by the P_app_ values. The involvement of passive diffusion, along with facilitated diffusion or efflux mechanisms, in the uptake and transport of xanthophylls was proposed. Further exploration of the transport mechanisms revealed that SR-BI and NPC1L1 were involved in the apical transport of both free and esterified xanthophylls, as evidenced by significant inhibition upon specific inhibitor treatment. By contrast, ABCG5 expression was selectively increased in response to ZEA and LUT rather than ZDP and LDP. Additionally, no significant changes in junctional protein (ZO-1, claudin-1) expression were observed, suggesting that tight junction integrity remained intact and paracellular transport was not involved. Furthermore, endocytosis via clathrin- and caveolae/lipid raft-dependent pathways contributed to cellular uptake of free and esterified xanthophyll micelles, whereas macropinocytosis was not involved. These findings provide insights into the varied uptake and transport mechanisms of free (ZEA and LUT) and esterified (ZDP and LDP) xanthophylls. Nevertheless, further studies are needed to establish the direct functional roles of the transport-related pathways identified here and to clarify the molecular basis underlying the distinct behaviors of free and esterified xanthophylls beyond expression-level responses.

## Figures and Tables

**Table 1:** Apparent permeation rates and trans-shipment ratio of free and esterified xanthophylls after exposed to differentiated Caco-2 cell monolayers.

	Recovery (%)	P_app_ (×10^−6^ cm/s)		P_ratio_
		AP → BL	BL → AP	
ZEA-M	97.89 ± 0.47	7.61 ± 0.33 a	5.73 ± 0.03 a	0.75 ± 0.03 a
ZDP-M	92.70 ± 1.26	7.45 ± 0.49 a	5.26 ± 0.02 b	0.71 ± 0.05 ab
LUT-M	91.47 ± 0.31	7.08 ± 0.19 a	4.82 ± 0.01 c	0.68 ± 0.06 bc
LDP-M	94.62 ± 1.33	6.94 ± 0.23 a	4.45 ± 0.10 d	0.64 ± 0.02 c

## Data Availability

The data presented in this study are included in the article and Supplementary Materials. Further inquiries can be directed to the corresponding author.

## Supplementary Materials

The following supporting information can be downloaded at: https://www.mdpi.com/article/10.3390/nu18091389/s1. Figure S1: Particle size distribution (A) and average particle sizes (B) of the free and esterified xanthophyll micelles. Figure S2: Cytotoxicity assays of Caco-2 cells treated with free and esterified xanthophyll micelles. Figure S3: Establishment of a 21-day Caco-2 cell monolayer model.

## Abbreviations

The following abbreviations are used in this manuscript:

ZEA	Zeaxanthin
LUT	Lutein
ZMP	Zeaxanthin palmitate
ZDP	Zeaxanthin dipalmitate
LMP	Lutein palmitate
LDP	Lutein dipalmitate
SR-BI	Scavenger receptor class B type I
NPC1L1	Niemann–Pick C1-like 1
ABCG	ATP-binding cassette transporter G
TJ	Tight junction
ZO-1	Zonula occludens-1
BLT-1	Block lipid transport-1
EIPA	5-(N-ethyl-N-isopropyl)amiloride
ZEA-M	Micelles containing zeaxanthin
ZDP-M	Micelles containing zeaxanthin dipalmitate
LUT-M	Micelles containing lutein
LDP-M	Micelles containing lutein dipalmitate
CCK-8	Cell counting kit-8
TEER	Transepithelial electrical resistance
AKP	Alkaline phosphatase
AP	Apical
BL	Basolateral
Papp	Apparent permeability coefficient
Pratio	Efflux ratio
