# Supplementary material for: Mechanisms of Cell Uptake and Transport of Xanthophylls in the Caco-2 Cell Model
Source: Nutrients. 2026 Apr 28;18(9):1389. doi: 10.3390/nu18091389 (PMC13164628; doi:10.3390/nu18091389)
Supplement: Supplementary file 1 [file nutrients-18-01389-s001.zip › nutrients-4226727-supplementary.pdf]

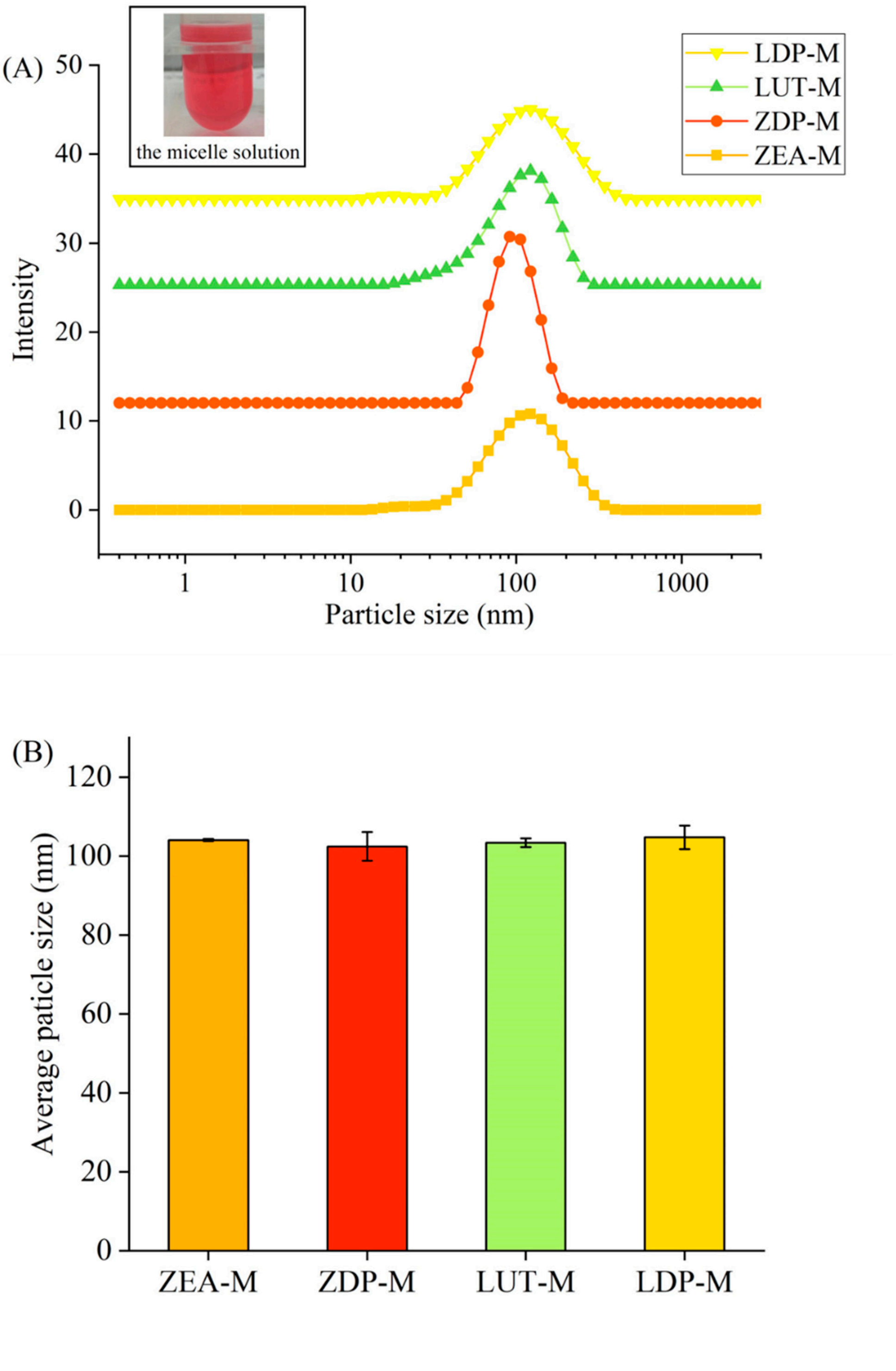

Supplementary Figure S1. Particle size distribution (A) and average particle sizes (B) of the free and esterified xanthophyll micelles. Data are expressed as mean ± SD, *n*=3. ZEA-M, ZDP-M, LUT-M, and LDP-M are micelles of zeaxanthin, zeaxanthin dipalmitate, lutein, and lutein dipalmitate, respectively.

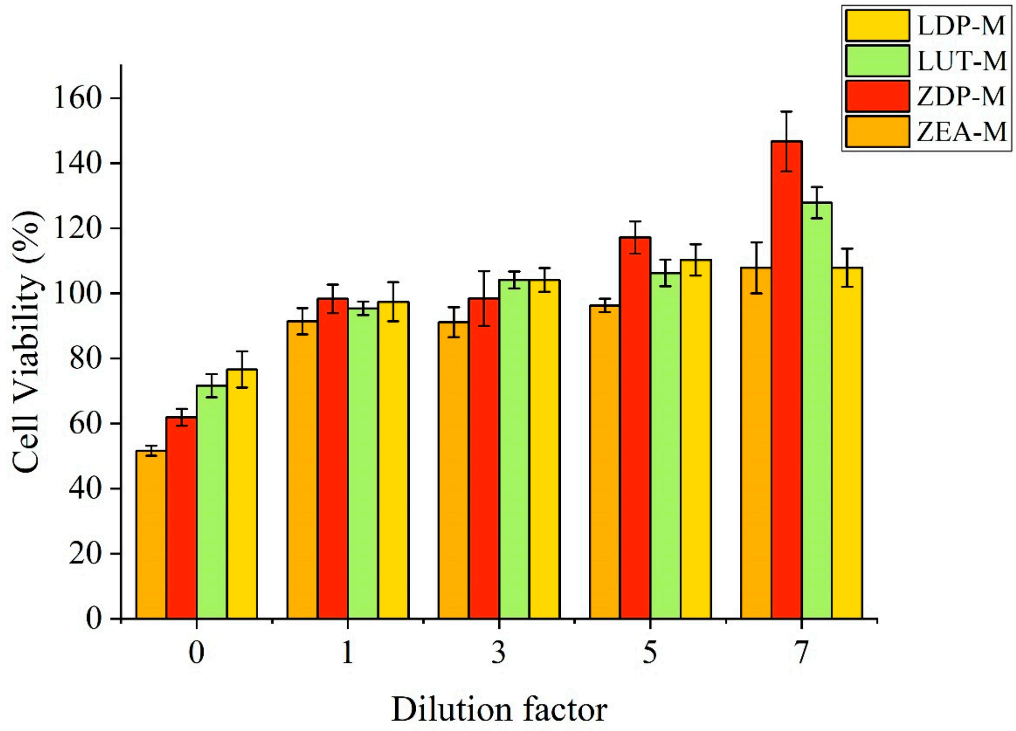

Supplementary Figure S2. Cytotoxicity assays of Caco-2 cells treated with free and esterified xanthophyll micelles. Dilution factors of 1, 3, 5, and 7 implied the micelles were diluted with DMEM medium at ratios of 1:1, 1:3, 1:5, and 1:7, respectively. The initial xanthophyll concentrations in ZEA-M, ZDP-M, LUT-M, or LDP-M were 1.84 ± 0.13 nmol/mL, 1.90 ± 0.08 nmol/mL, 1.92 ± 0.02 nmol/mL, and 1.86 ± 0.01 nmol/mL, respectively. Data were expressed as mean ± SD, *n*=5. ZEA-M, ZDP-M, LUT-M, and LDP-M are micelles containing zeaxanthin, zeaxanthin dipalmitate, lutein, and lutein dipalmitate, respectively.

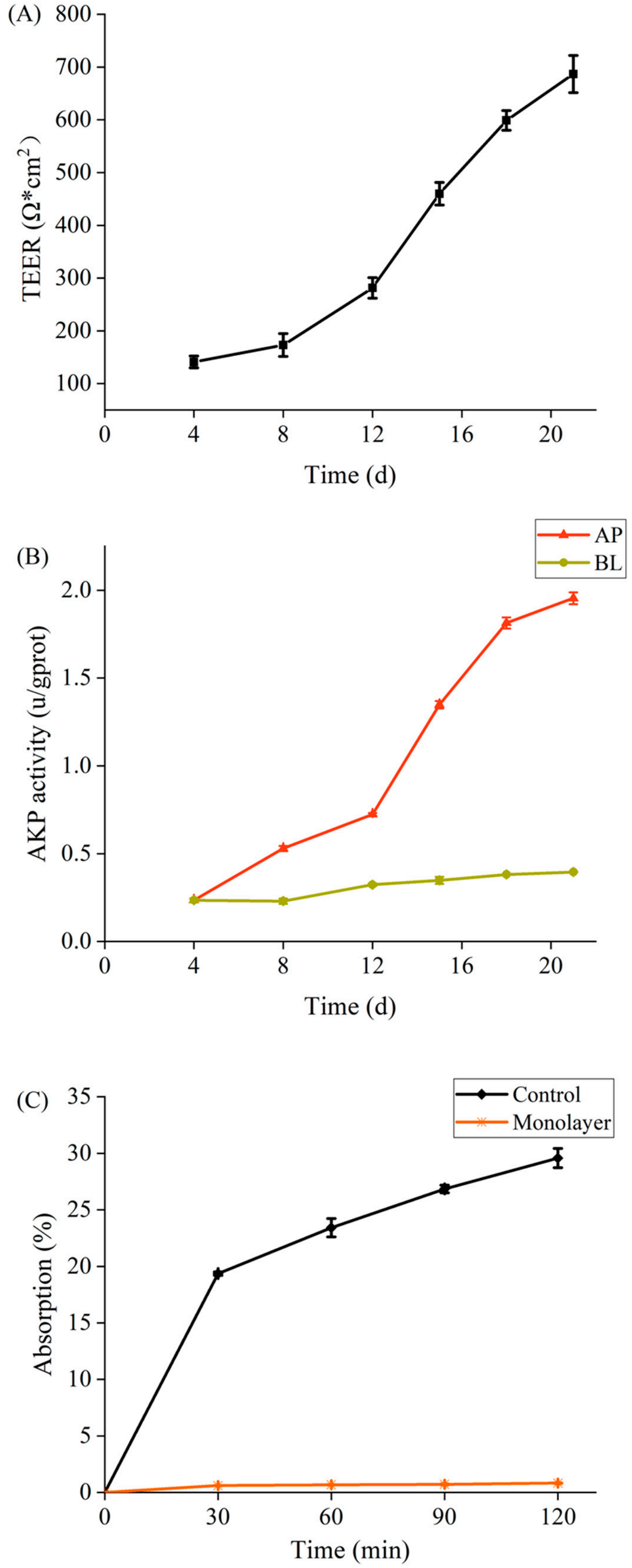

(A)
TEER (Ω*cm2)
800
700
600
500
400
300
200
100
0
4
8
12
16
20
Time (d)
(B)
AP
BL
AKP activity (u/gprot)
2.0
1.5
1.0
0.5
0.0
0
4
8
12
16
20
Time (d)
(C)
Control
Monolayer
Absorption (%)
35
30
25
20
15
10
5
0
0
30
60
90
120
Time (min)

Supplementary Figure S3. Establishment of a 21-day Caco-2 cell monolayer model. (A) Real-time monitoring of TEER value. (B) AKP activity at AP and BL sides. (C) Absorption of sodium fluorescein over time. AP: apical compartment of Caco-2 cell monolayer model; BL: basolateral compartment of Caco-2 cell monolayer model; Control: transwell cell culture chamber without Caco-2 cells; Monolayer: transwell cell culture chamber with Caco-2 cell monolayers. Data are expressed as mean ± SD, *n*=3.
